# Supplementary figures and images for: Revealing the dynamic whole transcriptome landscape of Clonorchis sinensis: Insights into the regulatory roles of noncoding RNAs and microtubule-related genes in development
Source: PLoS Negl Trop Dis. 2024 Jul 11;18(7):e0012311. doi: 10.1371/journal.pntd.0012311 (PMC11265684; doi:10.1371/journal.pntd.0012311)

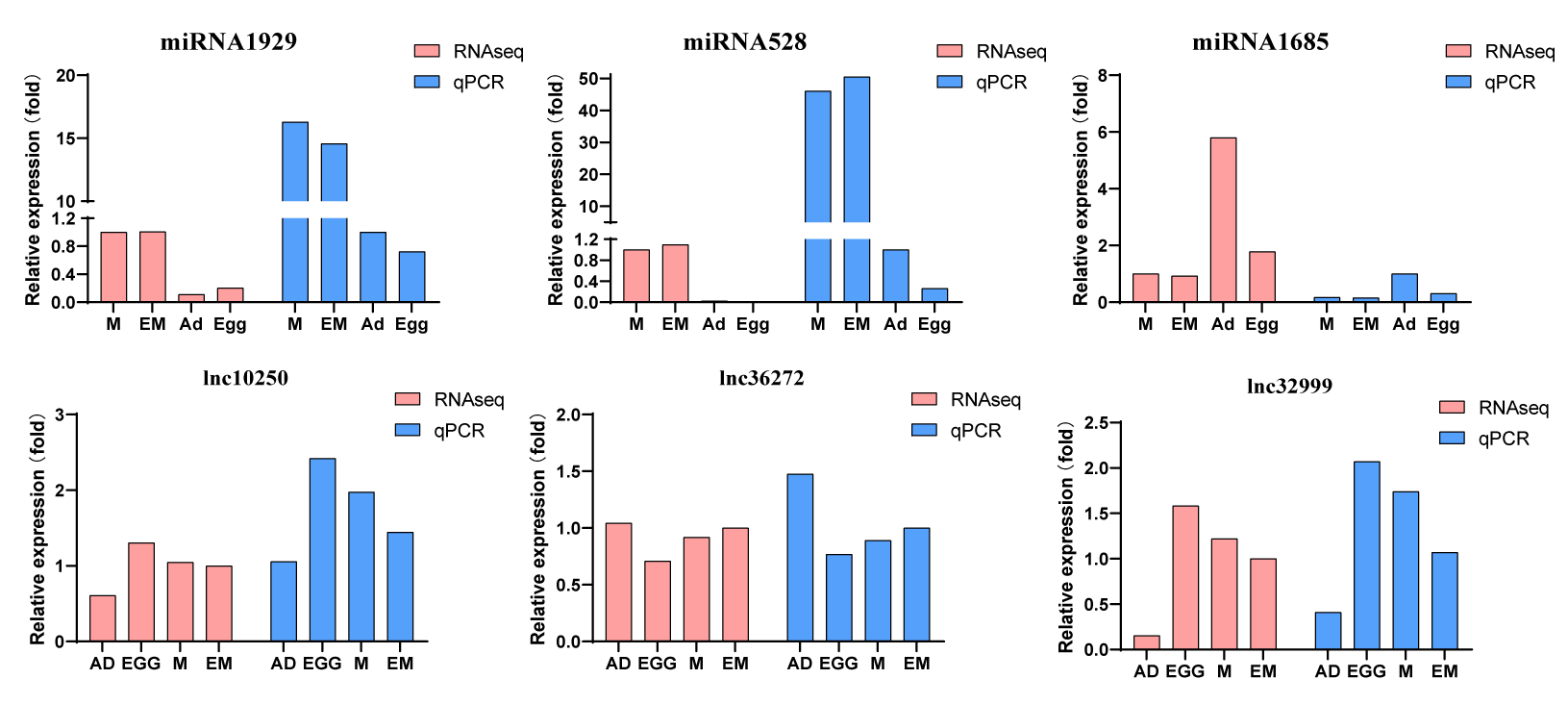

Supplement: S1 Fig — RT–qPCR results for the relative expression of miRNA and mRNA in egg (EGG), metacercariae (M), excysted metacercariae (EM) and adult (AD) of C. sinensis. (TIF) [file pntd.0012311.s001.tif]

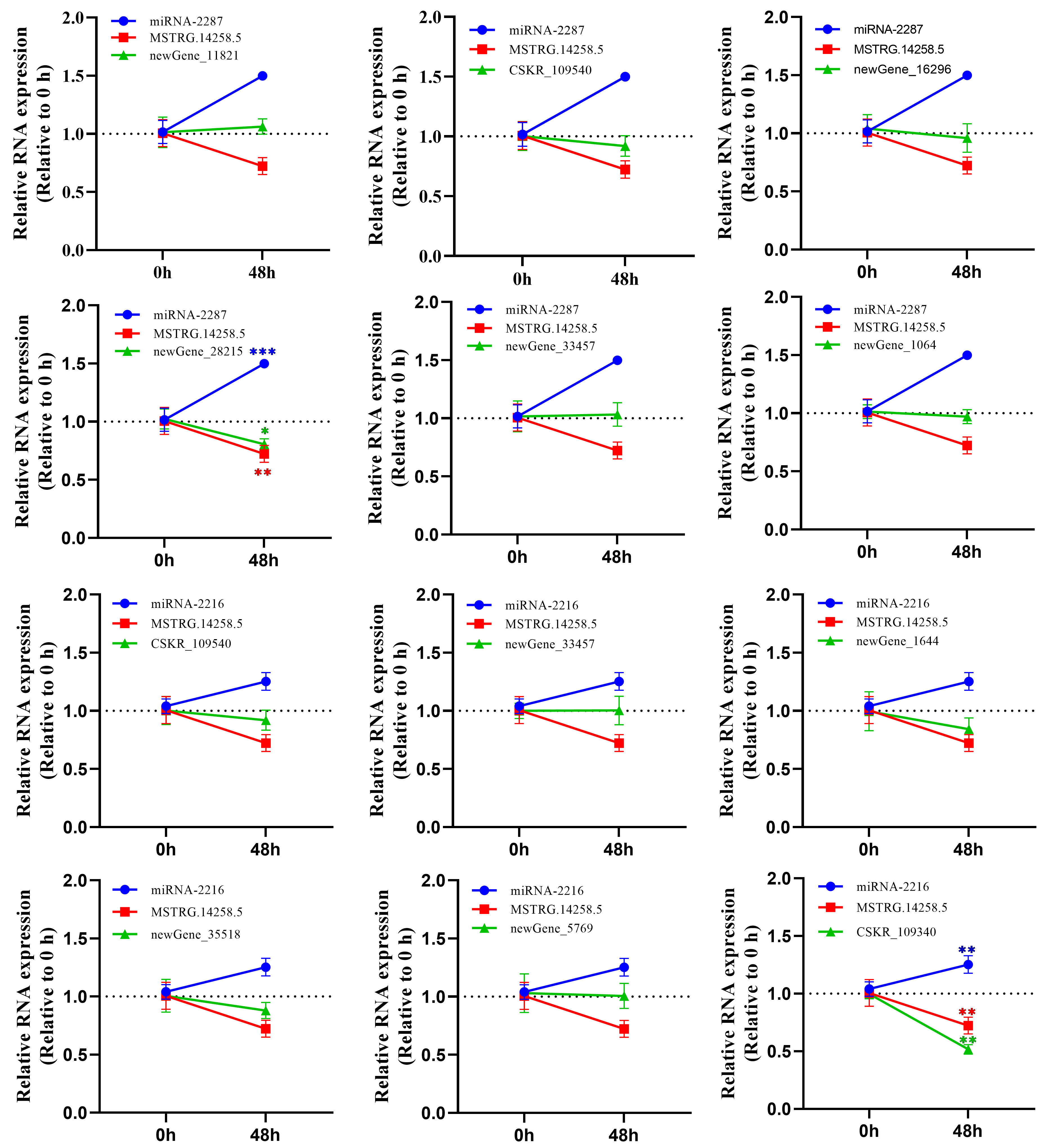

Supplement: S2 Fig — (TIF) [file pntd.0012311.s002.tif]
