# Supplementary material for: Revealing the dynamic whole transcriptome landscape of Clonorchis sinensis: Insights into the regulatory roles of noncoding RNAs and microtubule-related genes in development
Source: PLoS Negl Trop Dis. 2024 Jul 11;18(7):e0012311. doi: 10.1371/journal.pntd.0012311 (PMC11265684; doi:10.1371/journal.pntd.0012311)
Supplement: S1 Table — (DOCX) [file pntd.0012311.s003.docx]

|  | **Forward primer** | **Reverse primer** |
| --- | --- | --- |
| **U6** | TGGAACGCTTCACGAATTTGCG | GGAACGATACAGAGAAGATTAGC |
| **18S** | CATGCTTCCAGGTGCCCTTA | TACAAATGCCCCCGTCTGTC |
| **33KD** | AAAGACCCCACGAGCGAAAT | AAATGTCCTCCGTCTGCTGG |
| **DIC3** | TTCTTAAACCTGCGTCGCCT | TGAGAACCCTTGCGTCTGTC |
| **DLC2** | CGATATGGACCCACATGGCA | TAGGACCGCGTTGTTTCCAA |
| **DLC4** | GAGGGCTGAAGTTACCGAGC | CAATGAGTACATGCCACGCC |
| **HP290** | CCGGTGACTCAAAGGATGCT | TTTGCCAACGCGGTAAACAG |
| **HP9831** | AAGACCGCCGAAACCTCATT | CGTCTGTCTGGTTTCCCGAA |
| **WD63** | AGGTCCCATACTTCCAGGCT | GGTCATGCACAAAAACCGCT |
| **TAC1** | ATCCAAACGCACCATCCAGT | TTTGTGAGTCAGTCGAGCCC |
| **TB1** | GAACAGGAACAGCGCCTACT | AACATGGCGGCAAACTGTTC |
| **newGene11821** | GTCCAGCAGGCTTCAAGAGT | GTGGACCTGTACACGCTTGA |
| **CSKR109540** | CCTAGCTCTGCGGTGTGAAA | TGGGTGATTTGGATGGTCGG |
| **newGene16296** | ACTACTCAGCTTATCGCCGC | CCCATTGAGGTGTGCTGGAT |
| **newGene28215** | GACAACCAAATGCCCCCAAC | GTTTTCAAGCCACACCGGAC |
| **newGene33457** | ACTTTGGAGGCTACGCTCAC | ATGGGGAAGCATGAAACGGT |
| **newGene1064** | CACAATTTCGCTGTTGGGCA | CCACCTCGGACAGTTCAGTC |
| **newGene1644** | AGCCTCTTCATCAACACGGG | TATGACCCCCTCACCACCAA |
| **newGene35518** | CTCTGCGGTGGATGGTTTCT | TGGATCTCAGCGGACTGGTA |
| **newGene5769** | AGGACAGGAGGTAGAGAGCG | CCCGCACAGTATTCCAACCT |
| **CSKR109340** | TTGTCCTGTCTGCCAACCTG | TAGGAACGTGCGAGGTCAAC |
| **miRNA1929** | TGGAAGACTGGTGATATGTTGTT | Universal primer |
| **miRNA528** | CAACGATCACAAATCTCCATGT | Universal primer |
| **miRNA1685** | AACCCTGTAGACCCGAGTTTGA | Universal primer |
| **miRNA1107** | GCTTCTTGGAAGTTGGACTTGCG | Universal primer |
| **miRNA83** | GAACGGGTCAACAGTCATCTGA | Universal primer |
| **miRNA2287** | TCTTCACCCGGATGCAGATCTG | Universal primer |
| **miRNA2216** | AGCCTAGGGTCTAGTTCTTCT | Universal primer |
| **miRNA32** | TCCCGGGATTGTAAGTGGCGTA | Universal primer |
| **lnc10250** | ACCGAAGAACAGCTGTCAAGA | CTGGAAAACCCGTCACTCCT |
| **lnc36272** | TGTCCAGGTCGCCTAAGGAG | CTCGCCATTCACAGACGCTA |
| **lnc32999** | ATGTCGTGTGATGTCCCTCG | GGCGTTTAATGGCAAGAAAGC |
| **lnc14285** | CATCGGAGTCCTCATCCTGC | GTATCCTACGCATCACCGGG |

**Table S1. The *Clonorchis sinensis* primers that were used for qPCR.**

Note: 18S is 18S ribosomal RNA; 33KD is 33 kDa inner dynein arm light chain; DIC3 is dynein intermediate chain 3; DLC2 is dynein light chain 2; DLC4 is dynein light chain 4; HP290 is hypothetical protein CRM22_000290; HP9831 is hypothetical protein CRM22_009831; TAC1is tubulin alpha-1c; TB1 is tubulin beta-1; WD63 is WD repeat-containing protein 63; miRNA1929 is novel_miR_1929; miRNA528 is novel_miR_528; miRNA1685 is novel_miR_1685; miRNA1107 is novel_miR_1107; MiRNA83 is novel_miR_83; MiRNA2287 is novel_miR_2287; MiRNA2216 is novel_miR_2216; MiRNA32 is novel_miR_32; lnc10250 is MSTRG.10250.1; lnc36272 is MSTRG.36272.1; lnc32999 is MSTRG.32999.1 and lnc14285 is MSTRG.14258.5. The reverse transcription of miRNA employed the tail addition method, utilizing universal primers as reverse primers.
