# Supplementary material for: Revealing the dynamic whole transcriptome landscape of Clonorchis sinensis: Insights into the regulatory roles of noncoding RNAs and microtubule-related genes in development
Source: PLoS Negl Trop Dis. 2024 Jul 11;18(7):e0012311. doi: 10.1371/journal.pntd.0012311 (PMC11265684; doi:10.1371/journal.pntd.0012311)
Supplement: Attachment — Submitted filename: renamed_eaf22.docx [file pntd.0012311.s014.docx]

The authors describes research on *Clonorchis sinensis*, a food-borne parasite causing hepatobiliary diseases, including cholangiocarcinoma. Transcriptome sequencing and the chromosomal-level genome analysis were carried out to study RNA regulation during *C. sinensis* development. They identified thousands of differentially expressed RNAs across 37 developmental stages and highlighted the critical role of microtubule-related biological processes in the growth and development of *C. sinensis*. Two genes, DLC2 and DLC4, showed increased expression during development, and regulatory networks involving lncRNA-miRNA-mRNA and circRNA-miRNA-mRNA interactions were constructed. Overall, I find the study to be promising and potentially valuable in advancing our understanding of *C. sinensis* development. However, I do have some concerns regarding the balance between theoretical analysis and experimental data in the manuscript.

1. The author isolated *C. sinensis* from *Pseudorasbora parva* and then used rabbits as experimental subjects. Rabbits were infected with 500 metacercariae per rabbit, and parasites were collected at the 7th, 14th, and 56th days post-infection. How were the samples retrieved? It is suggested here that the author provide supplementary information regarding the method used to isolate parasites from rabbits.

2. The authors focus on bioinformatics analyses in the manuscript, while the presentation of experimental data is comparatively limited. While the use of high-throughput sequencing and bioinformatics tools is essential for gaining insights into transcriptomic profiles, it is equally important to support these findings with experimental validation. It would be beneficial to complement these findings with experimental validation. For example, the study could benefit from functional experiments such as gene knockout, knockdown, or overexpression studies to directly assess the roles of microtubule-related genes and non-coding RNAs in *C. sinensis* development. Including such experimental data would provide a more comprehensive understanding of the mechanisms involved.

3. In the results section of this article, the authors merely present data without providing summaries and conclusions for each result. It is advisable to include concise 1-2 summaries of the conclusions drawn from each result.

4. It is worth highlighting the practical implications of these findings, including their potential applications in the diagnosis and treatment of *C. sinensis* infections. Given the significant impact of *C. sinensis* on human hepatobiliary diseases, the insights gained from this study could potentially contribute to the development of more effective diagnostic methods and therapeutic interventions for this parasitic infection. Discussing these practical applications will not only underscore the relevance of the research but also its potential to make a meaningful difference in public health.
