# Supplementary material for: Revealing the dynamic whole transcriptome landscape of Clonorchis sinensis: Insights into the regulatory roles of noncoding RNAs and microtubule-related genes in development
Source: PLoS Negl Trop Dis. 2024 Jul 11;18(7):e0012311. doi: 10.1371/journal.pntd.0012311 (PMC11265684; doi:10.1371/journal.pntd.0012311)
Supplement: Attachment — Submitted filename: Response toPNTD-D-24-00043.R1.doc [file pntd.0012311.s015.doc]

**Dear Drs. Aysegul Taylan Ozkan and Richard Bradbury,**

On behalf of all co-authors, I would like to thank you and the reviewers very much for favorable and positive comments, and constructive suggestions on our manuscript (MS) ID **PNTD-D-24-00043**. These comments and suggestions are very valuable for us to revise and improve the quality of our MS. We have revised and improved the MS strictly according to the reviewer’s comments and suggestions. We used the “tracked changes” mode in the WORD to show the revised/changed text and sentences in the revised MS. The MS included “**tracked copy**” and “**clean copy**” is uploaded. In the following, we detail our point-by-point responses to these comments and suggestions. We hereby resubmit the manuscript for your kind consideration of publication in the journal of ***PloS Neglected Tropical Diseases***, and hope it is acceptable for publication in the journal.

**Responses to editors and Reviewers’ Comments Point by Point:**

**Reviewer #1:**

**Comment 1:** Minor revision.

The author isolated *C. sinensis* from *Pseudorasbora parva* and then used rabbits as experimental subjects. Rabbits were infected with 500 metacercariae per rabbit, and parasites were collected at the 7th, 14th, and 56th days post-infection. How were the samples retrieved? It is suggested here that the author provide supplementary information regarding the method used to isolate parasites from rabbits.

**Response:** Thanks very much for the constructive suggestions on our MS. We added the detail information about how to isolate 7th, 14th and 56th days parasites from rabbits in the MS. The detail information is below:

On the 7th and 14th days after infection, the rabbits were euthanized, and the liver tissues were separated and washed three times with PBS. First, the liver was cut along the bile duct and slowly compressed toward the common bile duct from the edge of the liver. Second, the liver was cut into small pieces and placed into a dish containing PBS. The larger tissue blocks were removed, and the parasites were collected under a stereomicroscope. The rabbits were euthanized on the 56th day after infection. Afterwards, the liver was separated and washed three times with PBS, and the liver was slowly compressed toward the common bile duct from the edge of the liver. Subsequently, the worms were squeezed out of the common bile duct and collected.

**Comment 2:** Major revision for results.

**Q1:** The authors focus on bioinformatics analyses in the manuscript, while the presentation of experimental data is comparatively limited. While the use of high-throughput sequencing and bioinformatics tools is essential for gaining insights into transcriptomic profiles, it is equally important to support these findings with experimental validation. It would be beneficial to complement these findings with experimental validation. For example, the study could benefit from functional experiments such as gene knockout, knockdown, or overexpression studies to directly assess the roles of microtubule-related genes and non-coding RNAs in C. sinensis development. Including such experimental data would provide a more comprehensive understanding of the mechanisms involved.

**Response:** Thanks very much for constructive suggestions, and we revised accordingly. Based on the suggestion, we have conducted an experiments to demonstrate the regulation network of long non-coding RNA in the growth process of *C. sinensis*. Through this experiment, we found that lncRNA (MSTRG.14258.5)-miRNA (novel_miR_2287)-mRNA (newGene_28215) and lncRNA (MSTRG.14258.5)-miRNA (novel_miR_2216)-mRNA (CSKR_109340) changed after lncRNA MSTRG.14258.5 was interfered, which are consistent with the regulation of ceRNA. In the future, we will also do more work to study the specific mechanisms of lncRNA during growth and development.

**Q2:** In the results section of this article, the authors merely present data without providing summaries and conclusions for each result. It is advisable to include concise 1-2 summaries of the conclusions drawn from each result.

**Response:** Thanks very much for the kind comments on our MS, and we revised according to your suggestion.

**Comment 3:** Minor revision for conclusion.

It is worth highlighting the practical implications of these findings, including their potential applications in the diagnosis and treatment of *C. sinensis* infections. Given the significant impact of *C. sinensis* on human hepatobiliary diseases, the insights gained from this study could potentially contribute to the development of more effective diagnostic methods and therapeutic interventions for this parasitic infection. Discussing these practical applications will not only underscore the relevance of the research but also its potential to make a meaningful difference in public health.

**Response:** Thanks very much for constructive suggestions, and we added more discussion about the practical applications, givosiran, as siRNA drugs in acute hepatic porphyria [1]. Thus, we can interfere with lncRNAs or circRNAs that play a key role in the developmental stages of *C. sinensis* through the RNAi to achieve the therapeutic goal of treating *C. sinensis*. In addition, we have added related discussion according your suggestion.

**[1]** Syed YY. Givosiran: A review in acute hepatic porphyria. Drugs. 2021; 81(7): 841-848. http://doi.org/10.1007/s40265-021-01511-3. PMID: 33871817.

**Comment 4:** Minor revision for Editorial and Data Presentation Modifications.

**Response:** Thanks very much for the “Minor revision”.

**Comment 5:** The authors describe research on *Clonorchis sinensis*, a food-borne parasite causing hepatobiliary diseases, including cholangiocarcinoma. Transcriptome sequencing and the chromosomal-level genome analysis were carried out to study RNA regulation during *C. sinensis* development. They identified thousands of differentially expressed RNAs across 4 developmental stages and highlighted the critical role of microtubule-related biological processes in the growth and development of *C. sinensis*. Two genes, DLC2 and DLC4, showed increased expression during development, and regulatory networks involving lncRNA-miRNA-mRNA and circRNA-miRNA-mRNA interactions were constructed. Overall, I find the study to be promising and potentially valuable in advancing our understanding of *C. sinensis* development. However, I do have some concerns regarding the balance between theoretical analysis and experimental data in the manuscript.

**Response:** Thanks very much for your review and affirmation about the manuscript. To make the data more convincing, we supplemented some experiments to prove the role of long non-coding RNA regulation networks in the growth process of *C. sinensis* (shown in Figure 7). In the future, we will also conduct more studies on the specific role of noncoding RNA in the growth and development process of *C. sinensis*. Thanks again for your suggestions.

**Reviewer #2:**

**Comment 1:** 1.Fig 1.

**Q1:** what are NIRI..... in panel d, Plz describe in legend.

**Q2:** Line 565, “on the” should added between “circular RNA” and “reference....”

**Q3:** Panel f should be redraw because that orange column was beyond the left boundary.

**Response:** Thanks very much for favorable comments and constructive suggestions on our MS.

**Q1:** NIRI02 is the WGS project for genome sequencing of *Clonorchis sinensis* (https://www.ncbi.nlm.nih.gov/datasets/genome/GCA_003604175.2/). In this WGS project, there are a total of 78 scaffolds (https://www.ncbi.nlm.nih.gov/Traces/wgs/NIRI02?val=NIRI02), and the final assembly compared two large (designed NIRI01200014 and NIRI0120005) and five small (NIR01200068, NIR01200048, NIR01200042, NIR01200017 and NIR01200006) chrome length scaffolds that containing 99.2% of all assembled scaffolds, and the specific content can be found in this article (High quality reference genome for *Clonorchis sinensis*, DOI: 10.1016/j.ygeno.2021.03.001). And we revised according.

**Q2:** Revised accordingly.

**Q3:** Revised accordingly.

**Comment 2:** Fig 2.

**Q1:** Line 572, in legend lncRNs and mRNs meaned lncRNAs and mRNAs?

**Q2:** Line 573 and Line 574, ”at” should be added before “different”

**Q3:** Panel c, EGG, M, EM, and AD meaned what? It should be interpreted in the legend.

**Response:** Thanks very much for favorable comments and constructive suggestions on our MS.

**Q1:** Yes, lncRNs and mRNs should be meaned lncRNAs and mRNAs, thanks your suggestion, we revised accordingly.

**Q2:** Revised accordingly.

**Q3:** EGG is egg of *C. sinensis*, M is metacercariae of *C. sinensis*, EM is excysted metacercariae of *C. sinensis* and AD is adult worm of *C. sinensis*. We added the explanation in the legend.

**Comment 3:** Fig 3, The comparision between what with what shouled be pointed out in Panel a, b,c and d.

**Response:** Thanks very much for the constructive suggestion on our MS, and we added the information of the comparisons in panels a, b, c and d. (a) egg::metacercariae, (b) metacercariae::excysted metacercariae, (c) excysted metacercariae::adult and (d) adult::egg.

**Comment 4:** Fig 4, “Enrichment of Gene Ontology Biological Process (GO-BP) of differential expression mRNAs in four developmental stages of C. sinensis” will be more suitable for the subtitle of the legend.

**Response:** Thanks very much for the constructive suggestion on our MS, and we revised accordingly.

**Comment 5:** Fig 5, The abbreviations of 33KD, DIC, DLC1, DLC2,....meaned what? It should be interpreted in the legend.

**Response:** The interpretations of 33KD, DIC, DLC1, DLC2,.... have been added in the legend of Fig 5, 33KD is 33 kDa inner dynein arm light chain; DIC3 is dynein intermediate chain 3; DLC2 is dynein light chain 2; DLC4 is dynein light chain 4; HP290 is hypothetical protein CRM22_000290; HP9831 is hypothetical protein CRM22_009831; TAC1is tubulin alpha-1c; TB1 is tubulin beta-1; WD63 is WD repeat-containing protein 63. Thanks very much for the suggestion.

**Comment 6:** Fig 6 and 7, “the more critical miRNAs (TOP5 degree) were marked in red” should be included in the legends.

**Response:** Thanks very much for the constructive suggestion on our MS, and we added accordingly.

**Comment 7:** In all figure legends, “Clonorchis sinensis” could be replaced by “C. sinensis”.

**Response:** Thanks very much for the constructive suggestion on our MS, and we revised accordingly in all figure legends.

**Comment 8:** Fig S1 legend should be provided.

**Response:** Thanks very much for the constructive suggestion on our MS, and we added the Fig S1 legend in our MS.

**Comment 9:** Why parasites were collected at the 7th, 14th, and 56th days post infection?

**Response:** Since the growth and development of *C. sinensis* is our main focus, we want to compare the RNA changes in early stage and adult stage. According to the life cycle of *C. sinensis, C. sinensis* usually is adult stage in rabbit after 28 days. We chose the 7th and 14th days is because *C. sinensis* has not yet become an adult in these days. As for why we didn't choose an earlier time, it is because the body of *C. sinensis* is very small and difficult to obtain before the 7th day. We tried to collect *C. sinensis* in the third day, but failed. We chose the 56th day is because it is now an adult stage. Based on these reasons, we chose the 7th, 14th and 56th days.

**Comment 10:** 47.6% (20/42) of mRNA transcripts are related to the cytoskeleton, including 6 tubulin alpha/ beta chains, 1 dynein intermediate chain, and 5 hypothetical proteins. The author randomly selected 8 mRNAs for qPCR validation. Why tubulin alpha/ beta chains were not included?

**Response:** We apologize for the neglect to tubulin alpha/ beta chains. In the revised version, we added the expression levels of tubulin alpha/beta chains of *C. sinensis* at the 7th, 14th and 56th days. Thank you for your suggestion.

**Comment 11:**

**Q1:** Line 453, 459, and 465, “dynein intermediate chain 2 and dynein intermediate chain 4” should be “dynein light chain 2 and dynein light chain 4”.

**Response:** Yes, “dynein intermediate chain 2 and dynein intermediate chain 4” should be “dynein light chain 2 and dynein light chain 4”, thanks very much for your constructive suggestions, and we revised accordingly.

**Q2:** The discussion about the gradually increase of DLC 2/4 is a little unconvincing.

**Response:** We apologize for the insufficient discussion. We have discussed again and revised in the MS. The new discussion is below:

Dyneins are microtubule-based molecular motors responsible for different intracellular transport processes and the regulation of cellular structures [1, 2]. Thus, as a component of the dynein motor complex, dynein light chain2 and 4 are essential to the transport of matter and energy. During the development of *C. sinensis*, demand for energy and matter is gradually increasing, meanwhile our result suggested that the expression level of dynein light chain2 and 4 increased correspondingly. In another study, researchers found that expression of dynein light chain of *Schistosoma mansoni* is developmentally regulated and localized to the tegument in the schistosomula, lung stage worms, and adult worms, but is not present in the cercariae or ciliated miracidia [3]. Thus, it is believed that DLC 2/4 may play a certain role in the growth and development of *C. sinensis*. We revised accordingly in the MS.

**References:**

[1] Goldstein LS and Yang Z. Microtubule-based transport systems in neurons: the roles of kinesins and dyneins. Annu Rev Neurosci. 2000; 23: 39-71. http://doi.org/10.1146/annurev.neuro.23.1.39. PMID: 10845058.

[2] Spiliotis ET, Kesisova IA. Spatial regulation of microtubule-dependent transport by septin GTPases. Trends Cell Biol. 2021; 31(12): 979-993. http://doi.org/10.1016/j.tcb.2021.06.004. PMID: 34253430.

[3] Hoffmann KF and Strand M. Molecular Identification of a *Schistosoma mansoni* tegumental protein with similarity to cytoplasmic dynein light chains. J Biol Chem. 1996; 271(42): 26117-26123. http://doi.org/10.1074/jbc.271.42.26117. PMID: 8824255.

**Q3:** Line 45-48, “This study suggested that through whole transcriptome sequencing the context of microtubule regulation might play a role in the development and growth of *C. sinensis*.” will be better than now.

**Response:** Thanks very much for constructive suggestions, and we revised accordingly.

**Comment 12:** The MS would be greatly improved if one ceRNA regulatory mechanism could be verified.

**Response:** Thanks very much for your constructive suggestions, and we revised accordingly. Based on the suggestion, we have conducted another experiment to demonstrate the regulation network of long non-coding RNA in the growth process of *C. sinensis*. Through these experiments, we found that lncRNA (MSTRG.14258.5)-miRNA (novel_miR_2287)-mRNA (newGene_28215) and lncRNA (MSTRG.14258.5)-miRNA (novel_miR_2216)-mRNA (CSKR_109340) changed after lncRNA MSTRG.14258.5 was interfered with, which are consistent with the regulation of ceRNA. In the future, we will also conduct more studies on the specific mechanisms of lncRNA in growth and development.

**Comment 13:** The language should be checked by native speaker.

**Response:** We have polished the language by the editing service to improve readability of the manuscript, and we really hope that the language has been substantially improved.
