# Supplementary material for: Revealing the dynamic whole transcriptome landscape of Clonorchis sinensis: Insights into the regulatory roles of noncoding RNAs and microtubule-related genes in development
Source: PLoS Negl Trop Dis. 2024 Jul 11;18(7):e0012311. doi: 10.1371/journal.pntd.0012311 (PMC11265684; doi:10.1371/journal.pntd.0012311)
Supplement: Attachment — Submitted filename: Response to PNTD-D-24-00043.R2.doc [file pntd.0012311.s016.doc]

**Dear Drs. Aysegul Taylan Ozkan and Eva Clark,**

On behalf of all co-authors, I would like to thank you and the reviewers very much for favorable and positive comments, and constructive suggestions on our manuscript (MS) ID **PNTD-D-24-00043R1**. These comments and suggestions are very valuable for us to revise and improve the quality of our MS. We have revised and improved the MS strictly according to the reviewer’s comments and suggestions. We used the “tracked changes” mode in the WORD to show the revised/changed text and sentences in the revised MS. The MS included “**tracked copy**” and “**clean copy**” is uploaded. In the following, we detail our point-by-point responses to these comments and suggestions. We hereby resubmit the manuscript for your kind consideration of publication in the journal of ***PloS Neglected Tropical Diseases***, and hope it is acceptable for publication in the journal.

**Responses to Reviewers’ Comments Point by Point:**

**Reviewer #1:**

**Comment 1:** The study design is appropriate and sufficient to address the stated objective. (Methods part)

**Comment 2:** The results are clearly and completely presented, of sufficient for clarity. (Results part)

**Comment 3:** The conclusions are supported by the data presented and public health relevance is addressed. (Conclusions part)

**Comment 4:** This paper not only described the expressions and interactions between mRNAs and ncRNAs of C. sinensis on the different stages of development, but also verified the interactions between lncRNA and miRNA. The sequencing data analysis and experiment designed were sufficient to support the object of the study, and the results were solid and comprehensive. (Summary and General Comments)

**Response to comments 1-4:** Thank you very much for your review of this manuscript, and we also appreciate your recognition of our research.

**Comment 5:**

1. Line 189, "sRNA" needs to be checked for accuracy.

2. Line 260, should it be for |r|?

3. Line266-267, should it be C. sinensis of 56 day?

4. Line396, what does 12 refer to? It needs to be clearly stated.

**Response:**

**Point 1:** "sRNA" should be "miRNA". Thanks very much for favorable constructive suggestion, and we revised accordingly.

**Point 2:** Thanks very much for favorable constructive suggestion, and we revised accordingly.

**Point 3:** Thanks very much for favorable constructive suggestion, and we revised accordingly.

**Point 4:** 12 was indicated the numbers of ceRNA networks. Thanks very much for favorable constructive suggestion, and we revised accordingly.

**Reviewer #3:**

I respect the efforts of the authors for this study, and the authors try to reveal the RNA characteristics of C. sinensis at four developmental periods through comparative transcriptomics, and construct the ceRNA network based on these data. However, the article is flawed and needs to be addressed and revised for clarification.

**Response:** Thank you very much for your review of this manuscript, and we have revised the manuscript according to your suggestions.

**Major comments:**

**Comment 1:** mRNA codes the protein and is the central of ceRNA network. So why DE-mRNA was not chosen for quantitative validation, what’s the reason?

**Response:** Due to limited research on noncoding RNA of *Clonorchis sinensis*, especially lncRNA and circRNA, we focused on noncoding RNAs in this study. Thus, when constructing the ceRNA regulatory network of *Clonorchis sinensis*, we pay more attention to the expression of noncoding RNA. However, we also noticed the expression changes of mRNA. Firstly, as shown in Figure 5, we identified 42 differentially expressed mRNA co-expressed with lncRNA, and selected some mRNA for quantitative validation (Figure 5b). Secondly, as shown in Figure 7, when verifying the regulation of ceRNA network in lncRNA MSTRG.14258.5, we also quantitatively validated the expression of mRNAs CsKR_28215 and CsKR_109340 (Figures 7e and 7f).

**Comment 2:** In Results section (L359): ‘MSTRG.14258.5, MSTRG.19374.4, and MSTRG.9295.1 were identified as related to microtubule regulation lncRNAs’. These three lncRNAs were only predicted to be related to microtubule regulation, not identified in molecular experiment. Similar description in L497-499, ‘The co-expression of lncRNAs and circRNAs with microtubule-related genes suggest their participation in the growth and development regulation of C. sinensis through ceRNA regulatory mechanism’. This conclusion is not justified by the fact that the authors only constructed a ceRNA network based on sequencing results and software predictions, and did not experimentally verify that microtubule-related genes are involved in the growth and development of C. sinensis through the ceRNA regulatory mechanism.

**Response:** We apologize for the lack of precision in these conclusions, and we have made changes in the revised manuscript.

1. "MSTRG.14258.5, MSTRG.19374.4, and MSTRG.9295.1 were identified as related to microtubule regulation lncRNAs" has been changed to "lncRNAs MSTRG.14258.5, MSTRG.19374.4 and MSTRG.9295.1 were speculated to be possibly related to microtubule regulation.".

2. "The co-expression of lncRNAs and circRNAs with microtubule-related genes suggest their participation in the growth and development regulation of *C. sinensis* through ceRNA regulatory mechanism" has been changed to "The coexpression of lncRNAs and circRNAs with microtubule-associated genes suggested that they may be involved in the growth and developmental regulation of *C. sinensis* through a ceRNA regulatory mechanism, but further verification is needed in the future.".

**Comment 3:** The graphs were not high enough resolution and they were too blurry.

**Response:** We have improved the image quality and hope it will meet your requirements. To improve the resolution of the image, we split Figure 6 into Figures 6 and 7. In addition, the original image can be downloaded from the link in the upper right corner of the review file, so that you can get a clearer picture.

**Minors:**

**Comment 1:** L4-5. The number of authors does not match. the number of authors is 10 in L4-5, while L25-26, the number of authors is 11, there is an extra “Xiaoxiao Ma” among the co-authors, and the contribution of “Xiaoxiao Ma” is not stated in L524 “Authors' contributions”.

**Response:** The number of authors is ten, “Xiaoxiao Ma” is not the coauthors, and we have thanked him in the Acknowledge part. Thanks very much for your careful review, and we have also revised.

**Comment 2:** L5. Wrong corresponding author name. L5. And L26, it was ‘Bin Tang‘, but in L20, it was written Bing Tang.

**Response:** The corresponding author name is Bin Tang. Thanks very much for your careful review, and we have also revised.

**Comment 3:** L126. ‘the dynamic whole transcriptome landscape of Clonorchis sinensis’, however, the number of samples and biological replicates were not stated in Materials and Methods.

**Response:** Thanks very much for your constructive suggestions, and we have also revised in Materials and Methods. The revised sentences is “The samples of 5,000 metacercariae (M, n = 3), 5,000 excysted metacercariae (EM, n = 3), 30 adult worms (AD, n = 3) and 100,000 eggs (EGG, n = 3) were rapidly frozen in liquid nitrogen and stored at -80 °C until use”. The number of sample was not consistent because of the inconsistent size of the worms at different stages.

**Comment 4:** L172. Reference genome. The full name, and version of reference genome is required.

**Response:** Reference genome is *Clonorchis sinensis* genome **(**GenBank: GCA_003604175.2; isolate: Cs-k2**),** <https://www.ncbi.nlm.nih.gov/datasets/genome/GCA_003604175.2/>. Thanks very much for your suggestions, and we have also revised in our manuscript.

**Comment 5:** L188.What’s the meaning of sRNA here, according to the context, it seems to be miRNA.

**Response:** Thanks very much for your suggestions, and we have changed “sRNA” to “miRNA”.

**Comment 6:** L201. The name of the package should be DESeq2, please check and verify.

**Response:** Thanks very much for your suggestions, and we have revised accordingly.

**Comment 7:** L210, The version of software Goseq, KOBAS (l218) is required.

**Response:** Thanks very much for your suggestions, and we have revised accordingly. The version of GOseq R package is version 1.24.0, and KOBAS is version 3.0.

**Comment 8:** L229. What is the specific role of albendazole, what is the connection between the results of albendazole treatment and the results of differentially expressed genes at different developmental periods in this study.

**Response:** Albendazole is a parasitic drug, and it can bind to β-tubulins, preventing their polymerization into microtubules, thereby corrupting the cascade of cell division at metaphase, which ultimately leads to the death of individual cells and eventually the death of the parasite[**1**]. In our results, we found many mRNAs related to microtubules were differentially expressed at different stages. Thus, we chose albendazole to treated our parasites, and observed the changes in microtubule related mRNAs, as shown in figures 5b and 5c. Our results indicated that albendazole affected the expression of microtubule related genes dynein light chain 2 and dynein light chain 4.

[1] Jacob J, Siraj A, Steel A, Tan G, Jarvi S. Evaluation of the mechanism of action of albendazole on adult rat lungworm (Angiostrongyluscantonensis). Exp Parasitol. 2022; 242: 108355. http://doi: 10.1016/j.exppara.2022.108355.

**Comment 9:** The number of sequencing groups should be explicitly stated in the methods; meanwhile, the L292 differential gene analysis, should state that these differential RNA molecules should be the total number of RNA molecules compared across groups.

**Response:** We have added the numbers of sequencing groups in the methods. In the L292 differential gene analysis, groups are four different stage parasites, including adults, eggs, metacercariae and excysted metacercariae of *C. sinensis*. Thanks very much for your suggestions, and we have revised accordingly.

**Comment 10:** L294. “|log2FC| > 1”, the correct one is in line204，“log2(Fold change) > 1”，2 is supposed to be subscripted in L294.

**Response:** Thanks very much for your suggestions, and we have revised accordingly.

**Comment 11:** L294. 1)The threshold P<0.001 used here is inconsistent with that in the Methods (L203, adjusted P value < 0.001); 2)there is a problem with the threshold line in the vertical coordinate of Figures 3a-d (especially b); 3)and it is not stated which two groups are being compared for the differential expression analysis of the different RNAs in Figures 3a-d. 4)If it is different developmental periods, then the volcano plots for differential expression analysis of each RNA should not be only one Figure, and the other results should be put in the supplementary file.

**Response:** Thanks very much for your suggestions, and we have revised accordingly.

1): Yes, “*P*<0.001” is should be “”adjusted *P* value < 0.001”

2): We have revised.

3-4): The differential expression analyses of the different RNAs in Figures 3a-d are four different stages. And we provide comparative data of differentially expressed genes for every two different periods in supplementary file in revised version.

**Comment 12:** Figures

Fig2c, 2d are labeled “***” and “ns”, but their meaning is not explained in the figure notes of Fig2.

Fig5c. Figure 5 is labeled “***” and “*”, but the title does not indicate what they mean.

**Response:** Compared with indicated groups, ns is for no significance, * is for *P* < 0.05, ** is for *P* < 0.01, *** is for *P* < 0.001. Thanks very much for your suggestions, and we have revised accordingly.

**Comment 13: Version problems.**

**Point 1:** L288. Fig.2E, L289. Fig.2e.

**Point 2:** L307, 313, 315. According to the context, it is supposed to be Fig. 3e, not 2e.

**Point 3:** L546. Questioning the authenticity of its animal ethics approval letter. L546, Ethical approval and consent to participate, ‘All mice were handled in strict accordance with the People's Republic of China Animal Ethical Procedures and Guidelines’, while in L133, 224, 225 state that the experimental animals in this study were ‘rabbits’, not mice in the animal ethics approval letter.

**Point 4:** L572. It was supposed to be mRNAs, not ‘mRNs’.

**Point 5:** L574. ‘at’ was omitted before ‘different developmental stages’.

**Point 6:** L593. It was adult and excysted metacercariae in C. sinensis in (b) in L591, and here it was adult and excysted metacercariae in *C. sinensis*. Please check and revise the group names clearly.

**Response:** The above errors (from point 1 to point 6) are errors in the original manuscript (PNTD-D-24-00043), which have been corrected in the R1 version (PNTD-D-24-00043R1). For example, in the first revised version (PNTD-D-24-00043R1), Fig.2e is just Fig.2e, and there is no Fig.2E. But, we still thank you for your valuable comments again.
